# C. Richard Conti—Lifelong learning: Advocacy and implementation

**DOI:** 10.1002/clc.23910

**Published:** 2022-09-01

**Authors:** Nanette K. Wenger

**Affiliations:** ^1^ Emory University School of Medicine Atlanta Georgia USA; ^2^ Emory Heart and Vascular Center Atlanta Georgia USA; ^3^ Emory Women's Heart Center Atlanta Georgia USA

In 1988, the World Conference on Medical Education published the Edinburgh Declaration^1^ addressing multiple concepts of medical education including the continuity of learning throughout life, shifting the emphasis from didactic methods to self‐directed and independent study. The Declaration advocated educational programs that included all health resources in the community, not hospitals alone, and provided an emphasis on the promotion of health and prevention of disease. The Declaration highlighted the need for educational systems that ensure the achievement of professional competence and social values, not merely the retention and recall of information. Viewed through the lens of 2022, the emphasis on greater social awareness and provision of services to promote the health of all people – not merely to deliver curative services to those who can afford them or to whom it is readily available – was unbelievably prescient.

We will recognize that as medical knowledge, skills, and social requirements for patient care rapidly evolve, clinicians must become lifelong learners to provide effective care for their patients.^2^ Davies characterized lifelong learning as “a continuously supportive process which invigorates and empowers people to attain all the knowledge, attitudes, and skills” they will require throughout their lifetime and apply them with confidence, creativity, and happiness.^3^

An early advance in lifelong learning was ACCEL, the Audio *Journal of the American College of Cardiology* for which Dr. Conti served as Editor‐in‐Chief from 2000 to 2010. This learning platform provided information from experts worldwide regarding current, clinically relevant, and evidence‐based information in cardiology. Dr. Conti honed his stewardship of this new educational modality, recognizing an editor's dual responsibility to the authors and the readers (listeners). Meetings of the ACCEL Editorial Board were exciting events – selection of the experts to be interviewed, the topics to be addressed, the learning needs of the audience–with emphasis on this new modality of learning: a 10‐min audio experience on audiotapes, for example, available on the drive to and from work. These precepts of lifelong learning were accompanied by background information in a detailed written summary, educational objectives, suggested readings, and at times knowledge assessment tests and evaluation questions.

Again, within the American College of Cardiology, Dr Conti was Chair of the Self‐Study Education Materials Committee that served to create ACCSAP, the ACC Self‐Assessment Program, even today a popular learning modality among cardiac clinicians worldwide. Dr. Conti led as an early adopter of how education/learning is evolving in the digital age, with traditional teaching methods now replaced by more innovative approaches. The modern learning methods and strategies have evolved from a teacher‐based system to a system of teacher‐learner collaboration, to the contemporary model where the approach is more learner‐centered. This transition was pioneered within the ACCSAP, which was designed to identify personal knowledge gaps, re‐enforce existing knowledge, and learn new information. ACCSAP educational materials are currently available in both text and video format, allowing the learner to acquire information either by reading or by watching/listening to presentations.

As Editor‐in‐Chief of *Clinical Cardiology* from 1988 to 2011, Dr. Conti addressed a very different national and international audience for lifelong learning through the traditional written media–but his Editorial Board meetings with worldwide leaders in cardiology again highlighted the rapidly evolving knowledge in clinical cardiology and the expansion to subspecialties including pediatric cardiology, cardiovascular surgery, echocardiography, and innovative imaging techniques, as well as the emerging contributions of clinical practice guidelines worldwide.

The COVID‐19 pandemic has both challenged and enlightened the landscape of local, national, and international scientific conferences. Whereas we all miss the interpersonal relationships and informal hallway encounters of in‐person meetings, virtual conferences have opened the potential attendee audience to huge populations unable to physically or financially attend major scientific conferences. The technology rapidly evolved to include not only didactic presentations, but discussion panels, chat rooms, interactive experiences, and to extend the teaching/learning experience to a world of webinars, podcasts, and the like. The individualized topics and methodology of learning; the subspecialized areas of cardiology, such as cardio‐oncology, cardio‐obstetrics, geriatric cardiology, and adult congenital heart disease now beckon novice and experienced clinicians alike to further their personal education. And we are rapidly adopting this multifaceted learning experience to extend to the education of our patients and their families as well.

Teaching and learning in the digital age have up‐ended the culture of teaching and learning–and all are acquiring the skills that best ensure our learning. Didactic teachers are no longer the custodians and conveyers of information. Contemporary learners require that teachers design learning experiences to enable all learners to productively engage with the available rich resources and learning activities. Some may be visual learners, others auditory learners, others practice or sample learners–and our choices may vary with time and topic. But we have the luxury of exploring the world of evolving information that will enable us to better deliver individual cardiovascular patient care and to improve the public health of our nation and our planet.
